# Thinking Structurally: A Cognitive Framework for Understanding How People Attribute Inequality to Structural Causes

**DOI:** 10.1177/17456916221093593

**Published:** 2022-08-18

**Authors:** Jamie Amemiya, Elizabeth Mortenson, Gail D. Heyman, Caren M. Walker

**Affiliations:** 1 Department of Psychology, University of California, San Diego; 2 Department of Psychology, Stanford University

**Keywords:** inequality, causal inference, structural causes

## Abstract

To make accurate causal inferences about social-group inequalities, people must consider *structural causes*. Structural causes are a distinct type of extrinsic cause—they are stable, interconnected societal forces that systematically advantage some social groups and disadvantage others. We propose a new cognitive framework to specify how people attribute inequality to structural causes. This framework is rooted in counterfactual theories of causal judgment and suggests that people will recognize structural factors as causal when they are perceived as “difference-making” for inequality above and beyond any intrinsic causes. Building on this foundation, our framework makes the following contributions. First, we propose specific types of evidence that support difference-making inferences about structural factors: *within-group change* (i.e., observing that disadvantaged groups’ outcomes improve under better societal conditions) and well-matched *between-group comparisons* (i.e., observing that advantaged group members, who have similar baseline traits to the disadvantaged group, experience more favorable societal conditions and life outcomes). Second, we consider contextual, cognitive, and motivational barriers that may complicate the availability and acceptance of this evidence. We conclude by exploring how the framework might be applied in future research examining people’s causal inferences about inequality.

Structural causes of inequality are stable, interconnected societal forces that systematically advantage some social groups and disadvantage others (Bonilla-Silva, 2015; Carmichael & Hamilton, 1967; Crenshaw, 1989; Haslanger, 2016; Hatzenbuehler, 2016; Jones, 1997; Rucker & Richeson, 2021; Salter et al., 2018). Research across the social sciences has documented how structural factors—which include societal institutions and cultural beliefs—contribute to inequalities in life outcomes between racial (Hoffman et al., 2016; Lawrence & Mollborn, 2017; Pierson et al., 2020; Reece & O’Connell, 2016; Roberts & Rizzo, 2021), gender (Bian et al., 2018; Cheryan et al., 2017; Herd et al., 2019; S.-J. Leslie et al., 2015; Yavorsky et al., 2015), socioeconomic (Browman et al., 2019; Duncan et al., 2017; Kraus, Torrez, et al., 2019; Laurin et al., 2019; Phillips et al., 2020), and LGTBQ (Hatzenbuehler et al., 2010; Raifman et al., 2017) groups and can be further exacerbated at their intersections (Cole, 2009; Crenshaw, 1989; Lei & Rhodes, 2021; Purdie-Vaughns & Eibach, 2008; Sidanius et al., 2018).

Despite this fact, people often fail to acknowledge structural causes of inequality. Instead, many people favor intrinsic explanations that emphasize traits such as ability or motivation (Cimpian & Salomon, 2014; Hunt, 2007; Kluegel & Smith, 1986; Mistry et al., 2012; Sigelman, 2012) or essentialist explanations that assume fixed innate differences between groups (Dar-Nimrod & Heine, 2011; Donovan, 2016; Panofsky et al., 2021). Although intrinsic factors may mediate structural influences, it is problematic to treat them as the sole root causes of inequality or as immutable properties (Cheryan et al., 2017; Donovan et al., 2019; Hetey & Eberhardt, 2018; Laurin et al., 2019; Rucker & Richeson, 2021; Spelke, 2005).

In addition to its importance for understanding human differences, recognizing structural influences has implications for how people reason about social policy and intervention. Disregarding structural causes can lead to false conclusions, such as assuming that individuals are solely to blame for their disadvantage or that societal interventions will have no effect for some groups (McCoy & Major, 2007; Piff et al., 2020; Rucker & Richeson, 2021; Soylu Yalcinkaya et al., 2017; Weiner et al., 2011).

Given the importance of acknowledging structural causes, a critical question becomes how people attribute inequality to structural causes. Although research has focused on people’s ignorance of or motivation to reject information about structural factors (Cimpian & Salomon, 2014; Jost, 2019; Rucker & Richeson, 2021), less work has detailed how people successfully make this causal link. In this article, we present a novel framework, the *difference-making framework of structural causal inference*, to describe this inferential process.

We organize the article as follows. First, we describe the central issue: The causes of inequality are not readily apparent, and people can infer different causes, namely intrinsic or structural. Second, we outline our framework, which is rooted in counterfactual theories of causal judgment and posits that people infer structural causes if they perceive that structural factors were “difference-making” for inequality. Third, we propose specific types of evidence that support structural causal inference and, critically, contextual, cognitive, and motivational barriers to the availability and acceptance of this evidence. We conclude by outlining future directions for research on causal inferences about inequality.

## Making Sense of What Causes Inequality

People start noticing inequalities between social groups early in life (Rhodes & Baron, 2019; Santhanagopalan et al., 2022; Shutts, 2015). For example, by at least 5 to 7 years of age, children are aware that White people tend to be wealthier than Black people (Elenbaas & Killen, 2016; Mandalaywala et al., 2020; Olson et al., 2012), that men tend to hold higher-status occupations than women (Liben et al., 2001; Miller et al., 2018), and that people of high socioeconomic status tend to have more desirable possessions (Dore et al., 2018; Peretz-Lange et al., 2022).

However, even when people agree that there is inequality, they may still disagree about its causes (Hetey & Eberhardt, 2018; Mijs, 2018; Peretz-Lange et al., 2021; Vasilyeva et al., 2018; Vasilyeva & Lombrozo, 2020). For example, in judging why there are more men than women in the field of engineering, one person may favor structural causes that are extrinsic to the groups (e.g., men have more societal support than women to participate in the field), whereas another person may privilege intrinsic causes (e.g., men are inherently smarter than women at math; for a discussion of various causal narratives, see Dennehy & Dasgupta, 2017). Both causal accounts explain why there would be systematic group differences, and people may differ in which account they find more compelling. Indeed, prior work has documented substantial variation in the extent to which children and adults endorse structural versus purely intrinsic causes of inequality (Diemer et al., 2019; Flanagan et al., 2014; Godfrey et al., 2019; Kluegel, 1990; Mistry et al., 2016).

## Structural Causes as a Unique Type of Extrinsic Cause

On its face, structural causes may seem synonymous with *extrinsic cause*s—that is, any causes that are external to the individual. However, structural causes are a distinct type of extrinsic cause: They are stable, interconnected institutional practices and cultural beliefs that consistently advantage some groups and disadvantage others (Bonilla-Silva, 2015; Haslanger, 2016; Jones, 1997; Rucker & Richeson, 2021; Vasilyeva et al., 2018; Vasilyeva & Lombrozo, 2020). For example, the G.I. Bill is a structural cause of racial wealth inequality in the United States because it was an institutional policy implemented in a way that consistently advantaged White Americans’ ability to build wealth while disadvantaging Black Americans (Perea, 2015). In contrast, random adverse events, such as some natural disasters, are not structural causes but rather are merely extrinsic causes that are more evenly distributed across the population (see also “fatalistic causes”; Mistry et al., 2012, 2016).

The distinction between structural and merely extrinsic causes is nontrivial because they constitute distinct patterns of reasoning (Hunt, 2002; Mistry et al., 2012; Vasilyeva et al., 2021; Weiss-Gal et al., 2009; Zucker & Weiner, 1993). For example, Vasilyeva et al. (2021) found empirical evidence that inferring structural but not merely extrinsic causes heightens the salience of social categories (e.g., gender) and leads reasoners to expect that inequality will persist over time. These causes also point to qualitatively different policy responses. Addressing a merely extrinsic cause of financial hardship in a neighborhood (e.g., an unexpected hurricane) would warrant a local intervention effort. In contrast, a structural cause, such as discriminatory housing policies, indicates that deeper institutional changes are needed (Bullock et al., 2003). Given that structural causes have distinct properties and downstream consequences for reasoning and intervention, we propose that a novel framework for describing structural causal inference is needed.

## Science of Inequality

Before we discuss structural causal inference, we note that the science on the actual causes of inequality suggests that social-group disparities are due to a complex interplay of structural and intrinsic factors. For example, structural constraints, such as fewer educational opportunities for lower-income children, can negatively impact their intrinsic motivation to succeed (Browman et al., 2019; Laurin et al., 2019), suggesting that structural barriers can affect individual-level barriers to success (see also C. Lewis, 2016). At the same time, early and relatively minimal group differences may promote structural responses that reinforce and strengthen group distinctions (see, e.g., Wood & Eagly, 2002).

Despite this complexity, we discuss structural causes as independent from intrinsic causes for two reasons. First, this simpler conceptualization maps onto previous empirical work on people’s causal beliefs about inequality, which has focused on their endorsement of structural versus intrinsic causes (Diemer et al., 2019; Rizzo et al., 2018; Vasilyeva et al., 2018; Vasilyeva & Lombrozo, 2020). Second, structural factors are typically less salient to people than intrinsic factors (Cimpian & Salomon, 2014; Rhodes & Mandalaywala, 2017), which motivates our focused analysis on how people successfully overcome barriers to structural thinking. That said, because our theoretical approach broadly applies to how people make causal inferences about inequality, it is also relevant for understanding intrinsic inferences.

## A Difference-Making Theoretical Framework of Structural Causal Inference

How do people attribute inequality to structural causes? The standard view in the literature from psychology and education is that people need to be made aware and accept that structural constraints exist (Cimpian & Salomon, 2014; Piff et al., 2020; Rucker & Richeson, 2021; Weisgram & Bigler, 2007). From an educational perspective, this standard approach would seek to increase people’s awareness of the structural constraints that certain social groups face (e.g., Nelson et al., 2013) and reduce the psychological threat that may prevent people from accepting this information (e.g., Adams et al., 2006).

We propose that the standard approach addresses a critical part of the structural-inference problem but does not address it entirely. Although people need to be aware of structural constraints to consider them potential causes of inequality (especially historical policies that have blatantly discriminated against certain groups; see Bonam et al., 2019; Nelson et al., 2013), this awareness does not always lead to causal attribution. In judging any outcome, people can consider a number of candidate variables but identify some or even just one of the variables as the actual cause (Dweck & Leggett, 1988; Gilbert & Malone, 1995; Weiner, 1985). Consider this example from another domain: A student is trying to figure out why they failed a test. The student may be aware of many possible causes—their lack of preparation, poor skills, and an unfair test—but ultimately settle on their lack of preparation as being the actual cause.

Applying this logic to inequality, people could be made aware of and consider structural factors but still privilege intrinsic factors as the cause of inequality (see Amemiya et al., 2022; Yang et al., 2022). Our framework seeks to address the second, critical part of structural inference: Once aware of structural constraints, what leads people to recognize them as causal? We propose that people must perceive that structural factors made a difference for inequality, above and beyond any intrinsic causes. We next describe the theoretical foundation for our framework: counterfactual theories of causal judgment.

### Counterfactual theories of causal judgment

Counterfactual theories propose that people make causal judgments (i.e., *c* caused *e*) by comparing the known outcome, *e*, to a relevant alternative situation in which *c* had not occurred (Gerstenberg et al., 2021; D. Lewis, 1973; Mackie, 1974; Woodward, 2005). If the comparison reveals that the target variable is difference-making, such that the actual and counterfactual outcomes are different, people tend to endorse the variable as causal.^1^ To generate the counterfactual outcome, people intervene on the target variable, either through direct or simulated manipulation (Gerstenberg et al., 2021; Halpern & Hitchcock, 2015; Woodward, 2005). In our example above, the student may choose to study more for the next test to observe whether studying makes a difference in their performance. They may also rely on their prior knowledge that studying typically leads to better performance and simulate the counterfactual that studying would have improved this particular test score (for more on simulating counterfactuals, see Gerstenberg et al., 2021).

There is robust evidence that people reason counterfactually to facilitate causal judgments about a range of domains, including physical phenomena (e.g., Gerstenberg et al., 2017; Goddu & Gopnik, 2020; Sobel et al., 2004), common life events (e.g., Epstude & Roese, 2008), and, critically, societal issues (e.g., Lagnado & Gerstenberg, 2017; Nolan, 2013; Sunstein, 2016; Wendell, 2020). For example, Sunstein (2016) noted that counterfactual claims are central to how people assess culpability in the law: When judges and juries assess whether someone is liable for an outcome (e.g., a car accident), they reason counterfactually about whether the outcome would still have occurred if the individual had acted differently (e.g., paid more attention to the road; see also Branscombe et al., 1996). Likewise, people use counterfactuals to make judgments about whether certain events or individuals had a causal impact on the course of history (Nolan, 2013; Sunstein, 2016; Wendell, 2020). In one illustrative study, adolescents were asked to reason about Adolf Hitler’s importance for the Nazis’ rise to power in Germany (Wendell, 2020). Notably, 82% of participants spontaneously generated at least one counterfactual statement when addressing this question, with some participants inferring that Hitler was indeed difference-making (e.g., “If it hadn’t been for Hitler, the Nazis wouldn’t have seized power”), whereas others rejected Hitler’s importance because they believed that the outcome would have happened anyway (e.g., “Someone else could have taken his role before Hitler formed history in the way he did”).

### Application to structural causal inference

**Fig. 1.**
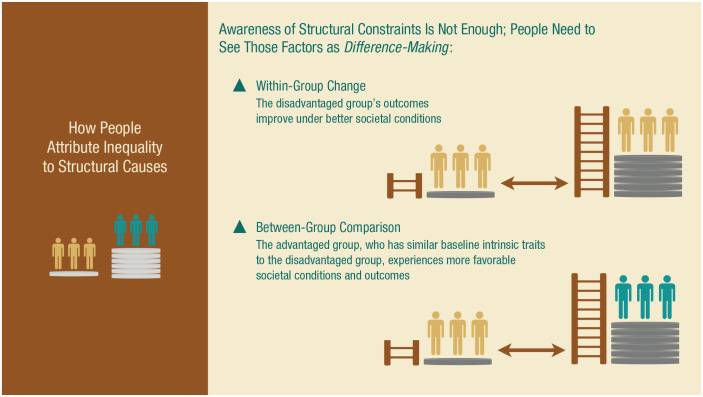
Overview of the difference-making framework of structural causal attribution.

Like other domains of reasoning, our framework contends that people reason counterfactually to assess the causal role of structural factors on inequality (for an overview of the framework, see Fig. 1). Here, the counterfactual question is: If the *societal structure* were different, would the level of inequality change? If people believe that removing societal constraints on disadvantaged groups would reduce inequality, they will recognize structural factors as causal. Consider the counterfactual comparison that author and journalist Isabel Wilkerson presents to argue for structural causes of racial inequality:

On those cotton fields were opera singers, jazz musicians, playwrights, novelists, surgeons, attorneys, accountants, professors, journalists. . . . We know that because that is what they and their children and now their grandchildren and even great-grandchildren have often chosen to become *once they had the chance to choose for themselves* [emphasis added]. (Wilkerson, 2017, 11:17)

Wilkerson’s argument rests on the counterfactual that Black Americans would have made significantly different life choices had they been provided with greater societal opportunities.

Critically, however, people can be aware of structural factors but ultimately reason that they are not difference-making and that intrinsic factors are the main cause of inequality. For this to be the case, the counterfactual outcome should reveal that inequality does not change when the disadvantaged group is given greater opportunities. Ex-Google employee James Damore used such counterfactual evidence in his 2017 antidiversity memo, “Google’s Ideological Echo Chamber.” He acknowledged sexism against women in STEM but emphasized that gender inequalities remain large across many societies and even become greater in egalitarian societies in which women presumably have greater opportunities (for a more nuanced interpretation of this phenomenon, see Breda et al., 2020). Counterfactual reasoning was central to Damore’s causal judgments, but he reached an intrinsic account because his counterfactual comparison suggested that structural factors do not make a difference for gender inequalities.

As demonstrated in these examples, perceptions of difference-making appear to be central to people’s structural inferences. Given that intervening on the target variable is a key part of this process (Gerstenberg et al., 2021; Halpern & Hitchcock, 2015; Woodward, 2005), it is important to specify how people “intervene” on structural factors, which are, by definition, complex and multifaceted. We posit that reasoners consider changes to parts of the social system (i.e., “soft interventions”; Eronen, 2020) and generalize these inferences to the broader societal structure. Indeed, human reasoners regularly build rich causal models of the world from the relatively sparse data that are available to them (Gopnik et al., 2004). In the next sections, we identify two types of evidence that serve as soft interventions on societal structures (we refer to this throughout the article as “interventional evidence”; see Griffiths et al., 2008). We posit that this evidence may be most efficiently obtained in the context of formal education (e.g., social studies) but also describe how people may encounter relevant instances in everyday life.

### Evidence type 1: within-group change

The first type of interventional evidence, *within-group change*, is the most conceptually straightforward and was captured in Wilkerson’s (2020) comparison of Black Americans’ life outcomes. Specifically, if people observe changes to parts of the societal structure (i.e., a soft intervention), followed by improvements in a disadvantaged group’s outcomes that narrows overall inequality between groups, they should infer that the structure was difference-making. This counterfactual information reveals that the inequality is mutable and, critically, varies as a function of structural factors (see also Gopnik et al., 2017; Heyman, 2009; Kelley, 1973). Within-group change also directly refutes essentialist explanations by showing that social groups’ properties are not fixed but responsive to the environment (Bastian & Haslam, 2006; Dar-Nimrod & Heine, 2011; Donovan et al., 2019).

The importance of learning about within-group change to infer structural causes aligns with Hetey and Eberhardt (2018), who proposed that Americans may accept structural causes of racial inequalities in the criminal-justice system if they are informed that crime in Black communities decreases when “tough-on-crime” policies are reformed or abandoned (e.g., easing minimum sentencing laws). This idea is an example of within-group change (i.e., changes in crime rates) in response to changes in the group’s structural constraints (i.e., laws and policing practices). Notably, children and adults spontaneously make the reverse prediction: When they construe group differences as being caused by societal factors, they expect to see within-group change across social environments (Noyes & Keil, 2020; Vasilyeva et al., 2018; Vasilyeva & Lombrozo, 2020).

Although people cannot directly observe within-group change on a large scale, they may observe changes in their own or others’ outcomes to make inferences about groups. For example, a racially marginalized student may notice improvements in their own academic performance when they transition to a more supportive educational context (for how people use this type of evidence when making causal judgments about individuals, see also Gopnik et al., 2017; Seiver et al., 2013). In one study of Black college students attending historically Black colleges and universities, a student made precisely this counterfactual observation: “The social aspect is completely different at Black colleges, which I’m very thankful for. I probably wouldn’t have the grades I have [elsewhere]” (Palmer et al., 2010, p. 95). People may use these individual observations to simulate the counterfactual outcome at the group level, for example, inferring that Black Americans, as a group, are also likely to improve in their academic outcomes when provided with greater educational opportunities (for the importance of personal experience in reasoning about societal issues, see also Kubin et al., 2021; Piff et al., 2020).

### Evidence type 2: between-group comparison

Given that it may be challenging to observe the same group under different societal conditions (see “Contextual Barriers” section), people may turn to other evidence that informs the disadvantaged group’s counterfactual outcome. We identify a second type of evidence, *between-group comparison*, that relies on the advantaged group who experiences fewer structural constraints as the counterfactual. The advantaged group offers insight into what may have happened to the disadvantaged group had they experienced the same favorable societal circumstances. To illustrate, when explaining women’s underrepresentation in STEM, people may consider the career outcomes of men, who typically have greater societal support to participate in STEM. People may then reason that women’s representation may look more like men’s had women been given the same support. Notably, this logic is embedded in the standard educational approach to structural inference, which informs students about the different societal constraints that groups experience.

At least for novel inequalities, there is evidence that the standard approach of using between-group comparisons promotes structural inferences (Brown & Bigler, 2004; Rizzo et al., 2018; Vasilyeva et al., 2018; Vasilyeva & Lombrozo, 2020). In one illustrative study by Vasilyeva et al. (2018), children and adults were introduced to an unfamiliar inequality in which girls play the game “Green-Ball” much less frequently than boys. Participants were then presented with a between-group comparison: Girls’ classrooms, but not boys’ classrooms, had physical barriers to playing Green-Ball (i.e., girls’ classrooms had a rule in which they had to throw a pebble into a small bucket to play, whereas boys’ classrooms had a larger bucket). This evidence led participants to explain the inequality in terms of girls’ structural barriers to playing the game. Our framework posits that participants’ structural inferences were supported by the fact that boys’ outcomes (i.e., they played Green-Ball at higher rates) illustrated what would have happened if girls’ environments afforded them to play Green-Ball.

To promote structural thinking about well-known inequalities, the standard between-group comparison approach needs to meet additional assumptions. Specifically, reasoners must perceive that the two groups are matched on all relevant intrinsic characteristics (e.g., abilities, motivation), such that the only difference between them is their societal constraints (for related theorizing, see Hatzenbuehler, 2016; Lantz et al., 2021; Lu et al., 2020). This assumption is easily met for novel inequalities that reasoners have no prior beliefs about (e.g., gender differences in playing Green-Ball) but not for inequalities that are widely stereotyped as being intrinsically based (e.g., gender differences in playing with dolls and trucks). If reasoners believe the compared groups are intrinsically different, they may reason that structural constraints are correlated only with intrinsic differences and are not causal for inequality (see Amemiya et al., 2022). Compelling between-group comparisons thus rely on the compared groups being carefully matched at baseline (e.g., men and women with similar baseline interest and ability in STEM).

Can reasoners make compelling between-group comparisons in their everyday lives? We propose that it is possible to the extent that reasoners can compare individuals from different social groups with similar traits. Socially diverse contexts that allow contact with many individuals from different social groups may afford such comparisons. For example, students in racially diverse schools may notice that Black and White students who engage in the same behaviors are treated differently by teachers (Yeager et al., 2017). As one Black adolescent observed, “I felt [my teacher] was being really racist to me because there was some white girl talking, and then I started talking, and then the teacher yelled at me” (Hope et al., 2015, p. 94). After making these observations about individuals, reasoners may infer that groups also experience differential treatment that leads to inequality above and beyond any intrinsic differences.

## Barriers to Structural Causal Evidence

Our framework has proposed two types of interventional evidence that can reliably promote structural thinking: within-group change and well-matched between-group comparisons. However, a theoretical framework should also account for why structural thinking is so rare (Cimpian & Salomon, 2014; Dunlea & Heiphetz, 2020; Hunt, 2007; Kluegel & Smith, 1986; Mistry et al., 2012). We contend that there are barriers to the availability and acceptance of both types of evidence. We consider contextual, cognitive, and motivational barriers in turn.

### Contextual barriers

Social contexts generally limit people’s ability to observe within-group change and between-group comparisons, both in everyday life and across history. A major limitation to observing within-group change, which entails observing groups’ responses to societal changes, is that societal structures tend to be stable rather than variable. Indeed, the societal institutions and cultural beliefs that contribute to inequality often persist across generations and settings (Bonilla-Silva, 2015; Haslanger, 2016; Hetey & Eberhardt, 2018). Two particularly salient examples are racial biases toward people of African descent and gendered societal roles for males and females. Moreover, societal changes that do occur (e.g., decreases in a society’s biases toward particular groups; see Charlesworth & Banaji, 2019) and any subsequent changes in inequality may be too slow or too small for people to readily detect. Thus, people may not observe within-group change in response to structural changes simply because it is uncommon for societal structures to shift dramatically.

With respect to making between-group comparisons, people must be in social contexts that are diverse enough to compare individuals who are from different social groups but have similar traits. The majority of people lack exposure to diverse social environments, particularly within the United States. Demography studies reveal high rates of segregation between social groups: For example, high-income American families tend to have low exposure to families from other socioeconomic backgrounds (Bischoff & Reardon, 2014; Reardon et al., 2018), and White American families often live in neighborhoods with other White individuals (Grigoryeva & Ruef, 2015). This issue may also come into play for gender: Although most people have exposure to individuals of the opposite gender at home or at school, they may have few close relationships with different-gender peers that would allow for insight into the other group’s experiences (Maccoby & Jacklin, 1987; Mehta & Wilson, 2020).

### Cognitive barriers

Our framework also suggests that there are cognitive barriers that can lead people to reject structural causal evidence, even when this information becomes available. As described in previous work, structural systems are complex and abstract, which may make structural information difficult to process (Cimpian & Salomon, 2014; Yang et al., 2022). This complexity may also explain why young children, who have relatively less knowledge about structural factors and limited cognitive resources to reason about such information, are especially unlikely to consider societal influences (Cimpian & Steinberg, 2014).

Critically, however, certain prior beliefs can impede structural inferences, even when the relevant evidence is presented in a way that is simple and accessible. In particular, preexisting essentialist beliefs may lead people to reject both types of interventional evidence that we have identified. As noted earlier, if people believe that groups are intrinsically different, then between-group comparisons in structural constraints are confounded by these baseline differences. Thus, someone with strong essentialist beliefs might reject a comparison between different groups’ societal opportunities because they assume that one group cannot be used to make counterfactual judgments about the other. Moreover, even if the groups were matched on important potential confounds such as interest and ability, strong essentialist beliefs may lead people to assume that deep, inherent differences still exist, thus compromising any further comparison.

Relative to between-group comparisons, essentialist beliefs may pose less of a challenge to evidence of within-group change given that within-group change directly refutes essentialist ideas that groups’ properties are fixed over time (Dar-Nimrod & Heine, 2011; Gelman, 2003). However, it is also possible that people with especially strong essentialist beliefs can accommodate within-group change in an essentialist framework. For example, people may assume that within-group change is also driven by genetic processes, such that groups’ genes are “evolving” differently (for a related misunderstanding, see Shtulman & Schulz, 2008).

### Motivational barriers

Motivated reasoning has been proposed as a key barrier to people’s structural reasoning (Phillips & Lowery, 2018; Piff et al., 2020; Rucker & Richeson, 2021). Social-psychological perspectives have highlighted people’s persistent denial of structural explanations, even after the causal link between structural factors and inequality is made obvious. This persistent denial stems from the psychological threat of structural explanations: They challenge many people’s preferred belief that societal systems are fair and call into question the assumption that life successes are rooted in merit alone (Jost, 2019; Kraus, Onyeador, et al., 2019; Phillips & Lowery, 2018, 2020). This threat is likely to be greatest for members of advantaged groups whose positions in society are justified by intrinsic explanations (Cech & Blair-Loy, 2010; Cozzarelli et al., 2001; Hartmann et al., 2009; Nelson et al., 2013; Panofsky et al., 2021; Phillips & Lowery, 2020) and to people with a social-dominance orientation (i.e., a preference for social hierarchies; Pratto et al., 1994). Here, however, we take a step back in the inferential process to consider how motivations may affect whether people infer the causal link between structural factors and inequality in the first place. Our framework, which seeks to complement existing social-psychological frameworks, proposes at least three mechanisms by which motivated reasoning affects the initial causal inference.

First, the type of counterfactual thinking needed to evaluate structural causes requires cognitive effort, in which people must represent actual inequality and then compare it to an alternative world to discern whether inequality would be different (Byrne, 2016). Individuals who are satisfied with the status quo (e.g., members of advantaged groups) may be unmotivated to engage in this reasoning process. Indeed, people are less likely to ask counterfactual “what-if” questions when they are satisfied with an outcome (Roese & Epstude, 2017), and they are less likely to search for causal explanations for outcomes that are familiar and pose no threat (e.g., Gendolla & Koller, 2001). Thus, some people may never seriously evaluate the causes of inequality, especially structural causes.

Second, even if people do engage in counterfactual reasoning to evaluate structural causes, they may strategically select or misrepresent counterfactual outcomes that support the conclusions they wish to make. For example, when asked to assess the U.S.’s progress toward racial equality, White Americans often used the past as the counterfactual reference point, which emphasizes the country’s progress, whereas racially minoritized people referenced ideal standards, which emphasizes the progress that has yet to be made (Eibach & Ehrlinger, 2006). Relatedly, recent work found that White Americans resist accepting information indicating that this comparison is false (i.e., that the United States has actually not made much progress toward racial equality from the past to the present; Onyeador et al., 2021).

A third mechanism is that people may strategically avoid certain situations that could influence the kinds of counterfactual comparisons they can make (for the variety of factors that influence the availability of counterfactuals, see also Byrne, 2016; Hitchcock, 2011; Kahneman & Miller, 1986). For example, people who are motivated to perceive the world as fair may avoid social contexts that could challenge this view (e.g., interacting with individuals outside of their racial group). Segregation from other groups may consequently limit the evidence available to make between-group comparisons of societal constraints (Merolla et al., 2011; Mijs, 2018). Overall, we propose that future work integrating social- and cognitive-psychological approaches could help inform how motivated reasoning impedes each part of the structural causal-inference process.

## Additional Directions for Future Research

In addition to providing a novel theoretical framework for understanding structural thinking, our approach raises new questions for empirical research. We have discussed causal inferences about structural factors as a broad category of causes, but an open question is whether some types of structural factors are more salient to people. We posit that people will be more accepting of structural causes that are concrete and deterministic, such as former policies at universities that completely forbade people of color and women from certain areas of study, compared with abstract and probabilistic structural causes, such as unwelcoming work environments in which underrepresented groups are negatively stereotyped (Bian et al., 2018; Master et al., 2016). The more abstract and probabilistic the constraint, the more it is possible to reason that the disadvantaged group could have acted differently if they had wanted to (e.g., persisted in STEM despite a hostile work environment), thus reducing its perceived causal power (see Walker et al., 2015).

Further, one general challenge for counterfactual accounts is to specify the alternatives that people spontaneously consider (Hesslow, 1988). In some domains, there tends to be agreement about which counterfactuals are most relevant (e.g., physical causation; Byrne, 2016; Gerstenberg et al., 2017). When reasoning about inequality, however, people may consider a much wider range of counterfactuals that serve various motivational and epistemic goals (Eibach & Ehrlinger, 2006; Kohler-Hausmann & Dembroff, 2022). For example, if people are interested in the effect of a specific policy, they may consider what happens to a disadvantaged group within the same societal context over time (e.g., changes in outcomes for American women under different social policies). However, if people are interested in the causal role of entire societal structures, they may care more about the group’s outcomes across different societal contexts (e.g., comparing the outcomes for women living in different countries).

Although we have largely focused on instances of within-group change for members of particular social groups (e.g., the changing life outcomes of Black Americans), it is also possible that learning how group categories have changed over time would bolster structural thinking (e.g., the evolving conceptualizations of race and gender throughout history; see Dembroff, 2018; Spencer, 2019). Recognizing that social-group categories are shaped by cultural conventions denaturalizes them and in turn may reduce the essentialist beliefs that impede structural inference. In support of this idea, a recent antiessentialist educational intervention that incorporated information about the changing nature of racial categories reduced people’s racial essentialism (Donovan et al., 2019).

There are also important questions regarding between-group comparisons. Here, we focused on comparisons that hold intrinsic factors constant (e.g., interest and ability) to determine whether structural factors are difference-making. However, people might also hold structural constraints constant to determine whether intrinsic properties of groups are difference-making. Specifically, if groups have unequal outcomes despite similar structural constraints, people may assume that the inequality is caused by characteristics inherent to the group (Vasilyeva et al., 2018; Vasilyeva & Lombrozo, 2020; Yang et al., 2022). Of course, this raises questions about whether two groups’ structural constraints are truly comparable. For example, scholars have argued that comparing Black Americans to other racial-minority groups who also experience racial discrimination (e.g., Asian Americans) is unfair because Black Americans’ societal oppression is qualitatively different (Godfrey et al., 2019; C. Lewis, 2016). However, we speculate that lay individuals may make such comparisons when they are available, even if they are invalid, when evaluating the causes of inequality.

Our framework may also inform current theorizing about belief polarization (for related theorizing, see Cook & Lewandowsky, 2016; Gershman, 2019; Jern et al., 2014) because exposure to structural causal evidence is constrained in part by an individual’s social context. Given the widespread segregation between social groups, it is likely that groups systematically differ in the evidence they observe, which may lead them to privilege different causes of inequality. Supporting this idea, Jefferson and colleagues (2021) found that Black and White Americans made different inferences about the most likely cause for a police shooting of a Black civilian: Black Americans more strongly inferred structural causes (i.e., police racial bias), and White Americans more strongly inferred individual causes (e.g., the civilian may have attacked the officer). In line with our account, the authors proposed that this pattern may be due, in part, to differences in each group’s exposure to racial bias in policing.

Finally, we note two important limitations that reflect broader limitations of the current literature. First, we drew from studies conducted predominantly in the United States (Cheon et al., 2020; Henrich et al., 2010), where one group (i.e., White Americans) possesses structural advantages in almost all aspects of society (e.g., economically, politically, numerically; Roberts & Rizzo, 2021). One interesting line of research would be to examine how people reason about inequality in societal contexts in which structural advantages vary across groups. For example, in Indonesia, Native Indonesians are a numerical majority and are well represented in the government but have significantly less wealth than Chinese Indonesians (Brown et al., 2018). It may therefore be more difficult for people to make clear structural causal inferences in the Indonesian context than the American context because societal forces are likely impacting groups in many directions.

Second, although we focused on inequalities between individual social groups (e.g., racial groups), research on people’s understanding of intersecting forms of societal oppression is critical (e.g., the unique forms of oppression that Black women experience; Cole, 2009; Crenshaw, 1989; Lei & Rhodes, 2021; Purdie-Vaughns & Eibach, 2008). An intersectional understanding requires more complex reasoning about inequality: Reasoners must be aware of the societal constraints that subgroups of people experience and need to keep at least four groups’ outcomes in mind rather than only two (e.g., Black women, Black men, White women, and White men). Although intersectional reasoning is more complicated, emerging research has shown that even young children have intersectional stereotypes for various subgroups (Jaxon et al., 2019; Lei et al., 2020), suggesting that people could also learn about intersectional forms of inequality.

## Conclusion

There is strong empirical interest in people’s structural thinking about inequality because it can be used to improve their understanding of human differences and their ability to make informed judgments about societal policies (Donovan et al., 2019; McLoyd, 2019; Mijs, 2018; Mistry et al., 2012; Phillips & Lowery, 2018; Piff et al., 2020; Rucker & Richeson, 2021). Moving forward, however, we note one important caveat to consider with respect to the consequences of structural thinking: Although inferring structural causes can reduce prejudice against disadvantaged groups, this is not always the case. Our argument is about inequality reducing once structural constraints are removed. Yet some people may argue that inequality is reduced only when structural constraints are added, and in this case it could increase prejudice. For example, attributing Black Americans’ and women’s increased representation in the workforce solely to affirmative-action policies is a structural causal inference but one that reinforces negative group stereotypes about ability (L. M. Leslie et al., 2014). Thus, much like essentialist explanations (see Peretz-Lange, 2021), inferring structural causes can both reduce and increase negative attitudes toward disadvantaged groups, depending on the phenomenon being evaluated.

Here, we focused on people’s structural thinking about inequality and proposed a novel cognitive framework that identifies the specific evidence to support structural causal inference, as well as unique contextual, cognitive, and motivational barriers to this inferential process. This line of inquiry may help to offer a path to more accurate lay understanding of persistent social inequalities.

## Transparency

*Action Editor:* Joshua Hicks

*Editor:* Laura A. King

## Notes

1. We note that there are additional approaches to causal judgment, such as *process theories* (Dowe, 2000; Shultz, 1982) and *associative theories* (Kruschke & Blair, 2000; Wasserman & Berglan, 1998), which emphasize features of actual events when making causal judgments. According to a process view, people consider the causal mechanism linking a candidate cause to the outcome (e.g., whether a physical quantity such as momentum transferred from Ball A to Ball B). Associative theories posit that reasoners infer causality on the basis of the strength of the association between two events (e.g., whether Ball A is present whenever Ball B passes through the gate). Despite their success in describing some aspects of causal cognition, these accounts do not capture the complexity of our intuitions that some causes “matter” whereas others do not (e.g., the chalk that is transferred from the cue stick to Ball A is not relevant for the ball’s movement).
